# When Paul Berg meets Donald Crothers: an achiral connection through protein biosynthesis

**DOI:** 10.1093/nar/gkae117

**Published:** 2024-02-26

**Authors:** Pradeep Kumar, Rajan Sankaranarayanan

**Affiliations:** CSIR–Centre for Cellular and Molecular Biology, Uppal Road, Hyderabad 500007, India; Academy of Scientific and Innovative Research (AcSIR), Ghaziabad- 201002, India; CSIR–Centre for Cellular and Molecular Biology, Uppal Road, Hyderabad 500007, India; Academy of Scientific and Innovative Research (AcSIR), Ghaziabad- 201002, India

## Abstract

Outliers in scientific observations are often ignored and mostly remain unreported. However, presenting them is always beneficial since they could reflect the actual anomalies that might open new avenues. Here, we describe two examples of the above that came out of the laboratories of two of the pioneers of nucleic acid research in the area of protein biosynthesis, Paul Berg and Donald Crothers. Their work on the identification of D-aminoacyl-tRNA deacylase (DTD) and ‘Discriminator hypothesis’, respectively, were hugely ahead of their time and were partly against the general paradigm at that time. In both of the above works, the smallest and the only achiral amino acid turned out to be an outlier as DTD can act weakly on glycine charged tRNAs with a unique discriminator base of ‘Uracil’. This peculiar nature of glycine remained an enigma for nearly half a century. With a load of available information on the subject by the turn of the century, our work on ‘chiral proofreading’ mechanisms during protein biosynthesis serendipitously led us to revisit these findings. Here, we describe how we uncovered an unexpected connection between them that has implications for evolution of different eukaryotic life forms.

## Graphical Abstract

**Graphical Abstract:**
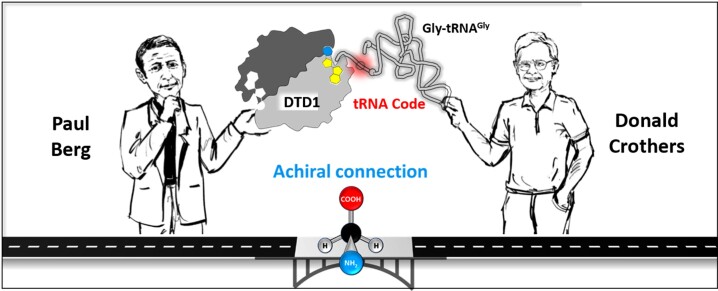

## Introduction

Faithful transfer of genetic information is crucial for the survival of living organisms. The errors associated with processes such as replication, transcription and translation are precisely controlled to a level that allows organisms to perform biological activities and adapt to varying environmental conditions. Unlike, replication and transcription, translation of genetic code from RNA to protein is a multistep process that begins with the addition of correct amino acid to respective tRNAs to form aminoacyl-tRNAs (aa-tRNAs) by aminoacyl-tRNA synthetases (aaRSs). Aa-tRNAs are then delivered to the ribosome by elongation factor thermo unstable (EF-Tu) for protein synthesis (1). Chemically similar pool of near-cognate amino acids presents a mechanistic challenge for the selection of correct amino acids by aaRSs. Quality control at the level of translation ensures uninterrupted supply of proteins with minimal errors for cellular functions. A number of molecular machineries starting from aaRSs to ribosome, in addition to various independent proofreading modules, are employed to keep the errors in check (1). Linus Pauling foresaw this more than half a century ago and determined the amino acid misselection rate of 1 in 5 between amino acids that vary by a single methyl group such as valine and isoleucine (2). Later, Loftfield and Vanderjagt used rabbit reticulocyte extract and experimentally calculated the translation error rate to be as low as ∼1 in 10^4^ (3). Within the same decade, Alan Fersht proposed a ‘Double-Sieve’ model which explained the proofreading function associated with aminoacylation reactions of aaRSs (4,5). According to this model, aminoacylation domain functions as a coarse filter that permits cognate and near-cognate amino acids while sterically rejecting bigger amino acids. The editing domain functions as a second sieve that finely eliminates similar near-cognate amino acids (4,5). The crystal structure of isoleucyl-tRNA synthetase (IleRS) provided the first structural and mechanistic evidence for the double-sieve model (6,7).

Errors during aa-tRNA synthesis by aaRS are of two types: amino acid misselection and tRNA misselection. The amino acid misselection can be further subdivided into two: near-cognate error and chiral error. Near-cognate errors include the selection of wrong amino acid similar in size and chemical properties (e.g. alanyl-tRNA synthetase (AlaRS) attaches non-cognate glycine and serine on cognate tRNA^Ala^ (8,9)). On the other hand, chiral errors involve mischarging of amino acid with opposite chirality (e.g. tyrosyl-tRNA synthetase (TyrRS) mischarges d-tyrosine instead of l-tyrosine (10,11)). A tRNA misselection error was identified recently wherein aaRS attaches cognate amino acid onto non-cognate tRNAs (e.g. archaeal and eukaryotic AlaRSs acylates cognate alanine to non-cognate tRNA^Thr^ (G4•U69) in addition to cognate tRNA^Ala^ (G3•U70) (12)). Such mischarged tRNAs are corrected by various proofreading modules employed by the translation apparatus (13,14). The proofreading modules attached to aaRSs are known as *cis* editors while free standing proofreaders are known as *trans* editors. If not corrected, non-cognate aa-tRNAs lead to the misincorporation of incorrect amino acids in proteome known as mistranslation. Mistranslation is potentially associated with protein misfolding and subsequent loss or gain of function resulting in generation of a statistical proteome. For example, even a partial loss of editing activity of AlaRS in mouse resulted in accumulation of misfolded proteins in neurons (15). These mouse models exhibited ataxia and neurodegeneration linked with loss of purkinje cells in cerebellum. The proofreading modules appended to the aaRSs have the capability to rectify the near-cognate error. However, the chiral error is only taken care by dedicated proteins termed as D-aminoacyl-tRNA deacylases (DTDs) (1,16,17). The chiral proofreading function is conserved across life forms. Bacteria and eukaryotes encode DTD1 whereas archaea and cyanobacteria employ completely different proteins DTD2 and DTD3, respectively, for the chiral proofreading function (18). All these versions of DTDs are of different evolutionary origins as they possess neither sequence nor structural homology amongst themselves (16,18). The widespread presence of DTD proteins in all the domains of life indicates that DTD activity is crucial and must have been instrumental in ensuring homochirality the last universal common ancestor (LUCA). Over the last decade, additional translation proofreading activities of DTD proteins apart from their chiral proofreading function were identified. DTD1 actively recycles glycine mistakenly attached to alanine specific tRNA and the discriminator base, 73rd nucleotide of tRNA, helps DTD1 in distinguishing cognate Gly-tRNA^Gly^ from non-cognate Gly-tRNA^Ala^ (19–21). DTD2 protects land plants by uniquely removing the *N*-alkyl-d-aminoacyl-tRNA adducts formed due to multiple physiological toxic aldehydes (22–26).

The evolution of genetic code is believed to be through an ambiguity-reduction mechanism (27). In similar lines, DTD proteins might have evolved their specific codes to select their substrates. DTD1 belongs to a family of DTD-fold proteins and shares structural homology with N-terminal *cis* editing domain (NTD) of threonyl-tRNA synthetase from archaea and animalia-specific tRNA deacylase (ATD) (13,28,29). Both DTD1 and ATD are shown to proofread chiral errors as well as near cognate errors, respectively (13,19,28), whereas NTD imparts near cognate proofreading activity in addition to showing binding for D-amino acids (29–31). The role of discriminator base in the biochemical activity of NTD and ATD needs further investigations to understand the significance of discriminator base in evolution of DTD-fold across life forms. It is interesting to note that DTD-fold executes both chiral as well as near cognate error correction through a hallmark RNA-based catalytic activity (32–34) and these vital biochemical activities must have emerged very early in evolution. Overall, the RNA-based catalysis and emergence of discriminator code based substrate selection by DTD1 signifies the transition from RNA world to RNA-protein hybrid world (35,36).

In this work, we focus on DTD1 along with its cross-talk with discriminator base of tRNA and how all life forms, eubacterial, archaeal and the two major forms of eukaryotes, have to resolve a ‘DTD1-Discriminator base conflict’, described later, not only for their emergence but also for their evolution (16,21,37,38). In the end, we summarize how the not so well recognized research findings from two of the pioneers of the modern molecular biology era have come together in a totally unexpected manner to reveal interesting facets of ‘Chiral selection mechanisms’ during protein biosynthesis.

### Paul Berg and DTD1

**Figure 1.**
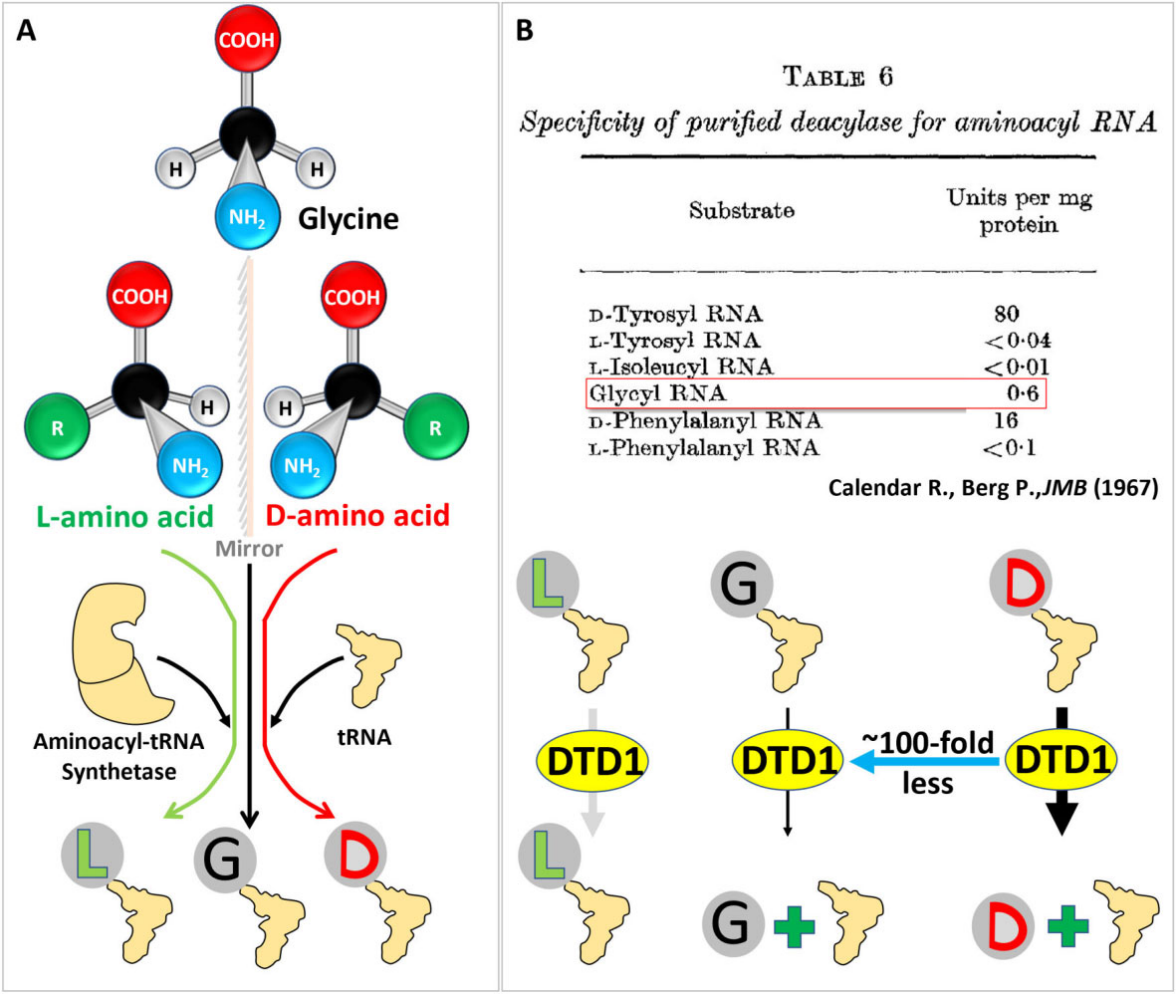
Paul Berg and DTD1. (**A**) Schematics showing the two chiral forms of amino acid, i.e. l- and d-amino acid, in addition to achiral glycine that are ligated to transfer RNA by aminoacyl-tRNA synthetases. (**B**) Table (Adapted from Calendar R., Berg P., JMB, 1967) and schematics showing the biochemical activity of d-aminoacyl-tRNA deacylase1 (DTD1) from bacteria on l- and d-aminoacyl-tRNAs and glycyl-tRNA originally observed by Calendar and Berg (17) (Here tRNA is referred to as RNA).

**Figure 2.**
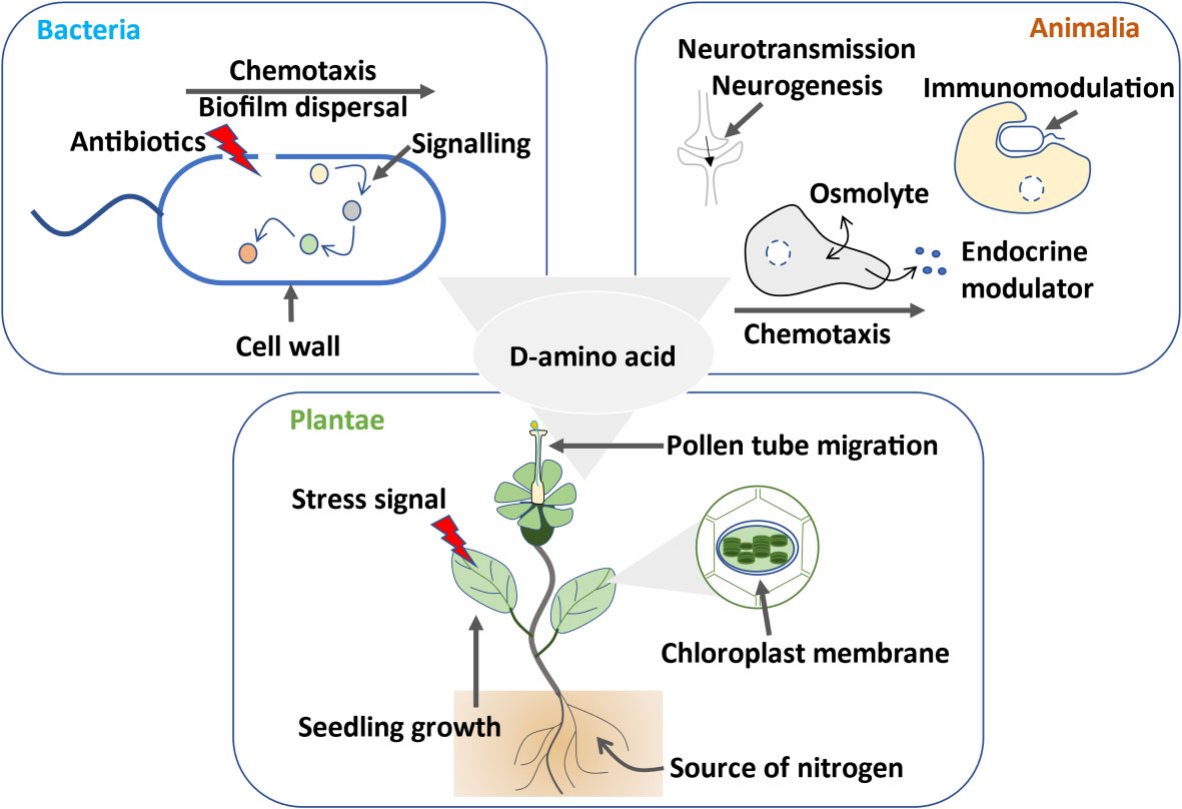
d-amino acids, though underappreciated, are ubiquitous and integral part of biological systems. Schematics showing the presence and physiological functions of d-amino acids in bacteria, animalia and plantae (52–60) (61–65).

Paul Berg began his career with the identification of acyl-adenylate formation by aminoacyl-tRNA synthetases (aaRSs) during protein biosynthesis (39–41). Although his work on recombinant DNA (rDNA) was a game-changer, he considered himself a biochemist at heart. When asked about his most impressive scientific achievement, he stated that ‘*Well curiously enough, I think my favorite experiments was the ones I did when I was a post doc’* and *‘In terms of personal satisfaction that was probably the most interesting, exciting, and rewarding experiment*.’ (42). Apart from rDNA work, he identified near-cognate errors associated with aaRS (43–47). He, along with his graduate student Richard Calendar, envisaged and showed that aaRS can charge D-amino acids on tRNAs (Figure 1A) (17), when d-amino acids were considered to be non-biological and possibly laboratory artifacts (48). He identified the DTD1 enzyme, a chiral proofreader, responsible for clearing these chiral errors made during the process of protein biosynthesis (Figure 1B) (17). DTD1 demonstrated high selectivity for d-aminoacyl-tRNAs (d-aa-tRNAs) (Figure 1B), providing new insights into biochemical processes that prevent d-amino acids from infiltrating into proteins (17). These findings went completely against the grain, as d-amino acids were, (and still are!) an ignored aspect of biology, with biochemical opinions being majorly dictated by l-chiral biased protein-based thinking until the turn of the century (Figure 2A)!

### d-amino acids: an underappreciated aspect of biology

d-amino acids are an integral part of biological systems. They are proposed to be present before the inception of life as they constituted almost half of amino acid composition in primordial soup similar to their l-enantiomers (49–51). Thereafter, one can envisage that d-amino acids got engrained in the diverse physiological processes of life. d-amino acids act as a source of nutrients and energy, signaling molecules, cellular defense, building blocks of antibiotics, components of cell wall, chemotaxis, neurotransmission, training the immune systems etc. (Figure 2) (52–58). Recent reports suggest that imbalance of d-amino acids is implicated in multiple neuropathological conditions (Alzheimer's disease, schizophrenia, amyotrophic lateral sclerosis and cognitive impairment), kidney disorders, cataract, cancer, atherosclerosis in humans; growth retardation, infection and pollination defects in plants; biofilm formation, spore generation and cell wall synthesis in prokaryotes (54,57,59–62). External application of d-amino acids act as a remedy for multiple above mentioned neuropathological conditions (54,57,59–61,63,64). Given the widespread presence of d-amino acids, these studies represent just the tip of the iceberg.

With a few anecdotal observations available until 1970s about the presence and physiology of d-amino acids, the field was uncharted due to the lack of suitable analytical techniques. Their widespread presence and physiological relevance started to unravel only with the advent of chiral chromatography towards the end of 20th century. Various d-amino acids like d-Ser and d-Asp were identified to be equivalent in their concentration to their l-counterparts in multiple organisms (65). D-amino acids have phenomenal physiological importance in life and are assigned with several crucial roles that were likely impossible for the l-enantiomers. However, it also has a flip side where their significantly higher concentrations present a challenge for the translation apparatus to maintain homochirality of the proteome. Identification of d-amino acid mischarging and an enzyme responsible for clearing mischarged d-aa-tRNAs, DTD1, by Paul Berg were pioneering observations that highlighted the importance of chirality during protein biosynthesis in biology (10,17). These observations went unnoticed for decades due to the lack of information about the physiological relevance of D-amino acids. The *in vivo* evidence for Paul Berg's observations started to appear after three decades when knockouts of DTD1, both in bacteria as well as in eukaryotes, showed growth defects in the presence of d-amino acids (11,66). This was a result of accumulation of d-aa-tRNAs inside cells which depleted free tRNA pools for fresh translation (67). Overall, d-amino acids, though underappreciated, are physiologically indispensable molecules for life which are integrated along with components like DTDs for maximizing physiological benefits and by minimizing their interference to key cellular processes such as protein biosynthesis.

### Donald Crothers and discriminator base

**Figure 3.**
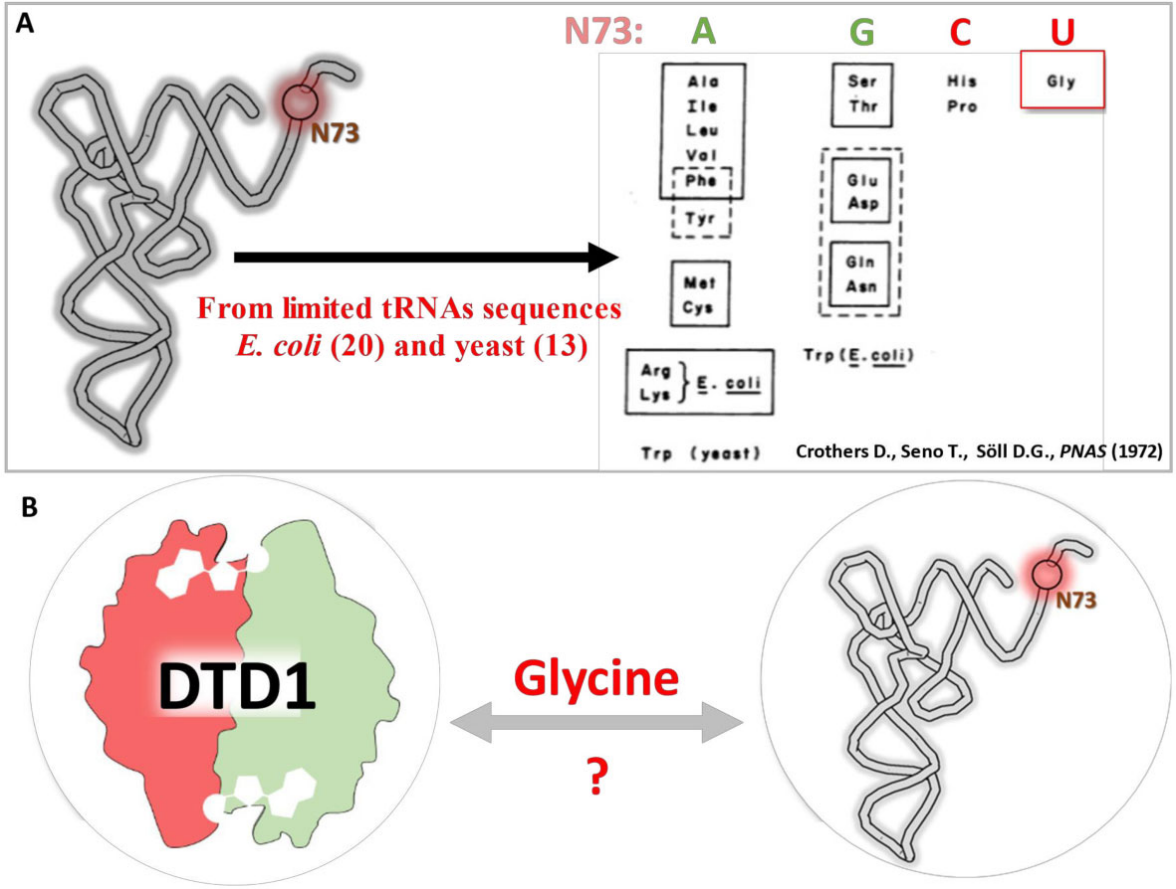
Donald Crothers and discriminator base. (**A**) Table (Adapted from Crothers D., Seno T., Söll D.G., PNAS, 1972) showing the discriminator base of tRNAs from *E. coli* and *S. cerevisiae* originally observed by Crothers, Seno and Söll (72) - The discriminator base of tRNA specific for glycine is highlighted in red. (**B**) Schematic showing achiral glycine linking the ‘chiral proofreader’ DTD1 with transfer RNA, the ‘Adaptor’ molecule in protein synthesis.

Similar to the accuracy in selection of cognate l-amino acid as substrate by aaRS, choosing the correct tRNA is also essential. The role of anticodon in decoding mRNA for protein synthesis was widely accepted in the 1960s (68–71) and the role of tRNA recognition by aaRS was first shown in 1966 (69). However, this was soon challenged by the discovery of suppressor tRNAs and the degeneracy of serine codons (69,72–76). As a result, researchers sought to identify a universal tRNA recognition mechanism by aaRS. Donald Crothers is well known for his contributions towards developing electrophoretic gel shift assays (EMSA) to study nucleic acid - protein interactions, and the role of nucleic acid sequence in structure and thermal stability (77). He, in close collaboration with Dieter Söll, a pioneering contributor in the area of protein synthesis, and his postdoc Takeshi Seno, came up with an elegant ‘discriminator’ hypothesis (72). While analyzing the limited number of tRNA sequences that were available till 1972 from *Escherichia coli* (20 tRNAs) and yeast (13 tRNAs), they noticed a striking correlation between the identity of the discriminator base and the chemical nature of the respective amino acid (Figure 3A) (72). Their astute observation that chemically similar amino acids tend to have a common discriminator site in tRNAs (i.e. adenine for hydrophobic, guanine for hydrophilic amino acids) (Figure 3A). It led them to envisage that such a system could have existed in a primordial scenario wherein chemically similar amino acids were selected purely based on discriminator base identity serving as an early ‘Discriminator Code’ (72). Later, multiple anticodon dependent as well as independent mechanisms, such as G3•U70 based selection by AlaRS (78,79) and variable loop-based selection by SerRS (80,81), were identified, which are possibly additions to a discriminator code selection for a primitive translation apparatus. Although Crothers′ hypothesis provided a potential explanation for the origin of amino acid selection, further research revealed that not all extant aaRSs use the discriminator base, and that the anticodon plays a major role in tRNA recognition (82,83). Therefore, the implications of the discriminator hypothesis were often overlooked.

### The enigmatic glycine

The DTD originally identified by Berg from eubacteria (17) (referred to as DTD1 here) is not conserved through evolution, but DTD function is present in all life forms highlighting the physiological importance of chiral proofreading (16). Furthermore, as mentioned above, it was identified that bacteria and eukaryotes possess DTD1, archaea and land plants contain DTD2 and cyanobacteria encode DTD3 (16). Calendar and Berg noted that while DTD1 was very selective for d-aa-tRNAs, it had a detectable activity on tRNA charged with the achiral amino acid glycine (Figure 1A) (17). However, the physiological rationale for the minimal activity on Gly-tRNA by DTD1 remained unexplained. Interestingly, the tRNA specific for glycine was an anomaly in the Crothers’ discriminator code hypothesis as well, wherein it was tabulated as possessing uracil as a discriminator base (Figure 3B) (72). These observations i.e. the uniqueness of tRNA^Gly^ in the discriminator hypothesis and the activity of DTD1 on glycyl-tRNA, went unnoticed and remained an enigma (Figures 1B, 3A, B).

### DTD1 meets discriminator base

**Figure 4.**
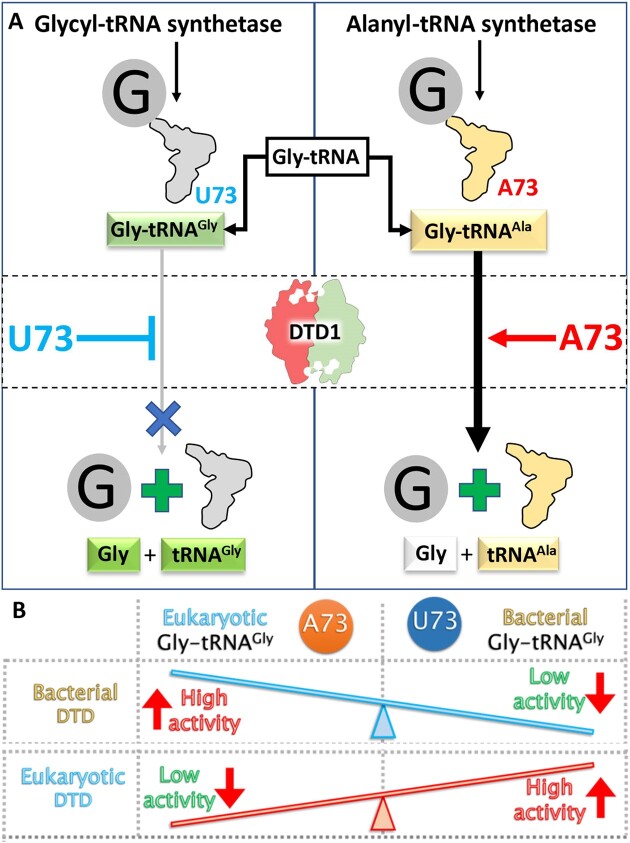
DTD1 meets discriminator base. (**A**) U73 as a discriminator base of tRNA^Gly^ acts as a negative determinant while A73 as a discriminator base of tRNA^Ala^ acts as a positive determinant for bacterial DTD1 to protect cognate Gly-tRNA^Gly^ and active recycling of non-cognate Gly-tRNA^Ala^, respectively. (**B**) Bacterial DTD1 show higher activity on A73 containing Gly-tRNA^Gly^ and eukaryotic DTD1 is hyperactive on U73 containing Gly-tRNA^Gly^.

Nearly half a century after these observations, our laboratory elucidated the mechanism of chiral proofreading by DTD1 wherein an invariant Gly-*cis*Pro motif from one subunit of a dimer captures the chiral center of the d-amino acid entering the other one (28). As a consequence, DTD1 operates by a strict l-chiral rejection mechanism and hence can act on achiral glycine (16,28). The misediting of glycine appeared to be the result of smaller substrate binding to a bigger pocket, as frequently observed in any protein-ligand interaction system. However, it was later identified that the action on glycine is not a design flaw but DTD1 doubles as a key factor in proofreading of glycine mischarged on tRNA^Ala^ by AlaRS (Figure 4A) (16,20). Glycine is mischarged at twice the rate of serine on tRNA^Ala^ (9), posing a more severe threat than serine. The solution to the glycine puzzle started to unravel when the activity of DTD1 was found to be ∼1000-fold more on Gly-tRNA^Ala^ compared to its cognate Gly-tRNA^Gly^ (Figure 4A) (19). Until then, DTD1 was believed to only recognize the amino acid chirality, and therefore was expected to be unaffected by incoming tRNA due to its activity on multiple d-aa-tRNAs. Finally, the physiological rationale for the weak activity of DTD1 on glycine observed by Berg had been deciphered.

This work further led us to identify the discriminator code anomaly that came out of Crothers’ findings as well. Remarkably, a single base switch i.e. discriminator base from pyrimidine to purine, in tRNA was found to be a major determinant that controls the activity of bacterial DTD1 by 100-fold. Bacterial DTD1 exhibits a higher preference for purine (A/G73) containing tRNAs over pyrimidine (U/C73) containing tRNAs (21). Such a negative determinant on the tRNA is an absolute necessity to protect the cognate Gly-tRNA^Gly^ (U73) from depletion, and to specifically cleave non-cognate Gly-tRNA^Ala^ (A73), thereby preserving glycine fidelity during protein biosynthesis in all bacteria (Figure 4A). Due to the unique co-evolution of DTD and discriminator base, this protection is crucial and far greater than that provided by EF-Tu (34). Thus, the discriminator-based substrate specificity code of DTD1 explains the selection pressure on bacterial tRNA^Gly^ to use U73 as an exception to the purine biased discriminator world observed by Crothers (21).

### Critical interplay of DTD-tRNA code for the emergence and evolution of eukaryotes

**Figure 5.**
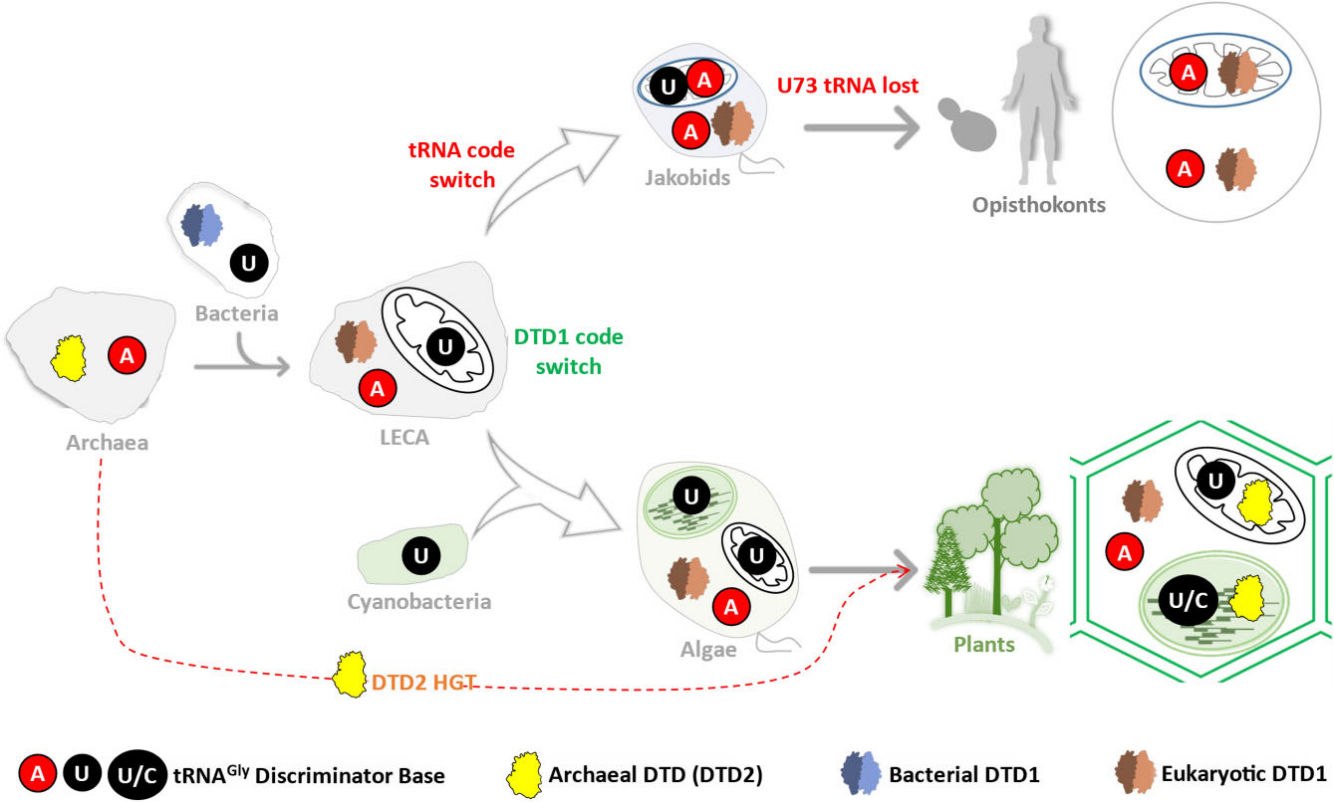
DTD-discriminator code evolution across life forms. Schematic showing the critical evolutionary dynamics of chiral proofreaders and tRNA^Gly^ discriminator base in different domains of life.

Eukaryotic DTD1s are similar to bacterial DTD1s (16). However, unlike their bacterial counterparts, they show higher activity on tRNA^Gly^ with pyrimidines as a discriminator base as they have to work with archaeal derived tRNA^Gly^s carrying adenine as discriminator base (Figure 4B) (Figure 5) (21). Therefore, the expression of eukaryotic DTD1 in bacteria and vice versa is toxic, wherein it depletes the Gly-tRNA^Gly^ of the host (37). This inverse discriminator base preference of bacterial and eukaryotic DTD1s presents an interesting evolutionary conundrum i.e. an enzyme performing the same biochemical function in two different domains of life is toxic when expressed in the other due to incompatible discriminator codes (Figure 4B) (37). This suggests a strong and selective co-evolution of tRNA^Gly^ discriminator base and the specificity of DTD1 across life forms.

The primary endosymbiotic theory proposes that eukaryotes emerged by the ingestion of a bacterium by an archaea around two billion years ago, with the bacterium transforming to become the mitochondria of the extant eukaryotic organisms. The encounter of A73-specific bacterial DTD1 and archaeal tRNA^Gly^ possessing A73 during the primary endosymbiotic event could have led to incompatibilities. Therefore, it was necessary for the eukaryotic DTD1 to evolve and optimize itself to ensure compatibility with the archaeal derived tRNA^Gly^ and prevent cell death. However, since mitochondrial tRNA^Gly^s are bacteria-derived and contain U73, eukaryotic DTD1 would be incompatible with them. This is where the switch of the mitochondrial tRNA^Gly^ discriminator base from U73 to A73 comes into play, thus protecting mitochondrial Gly-tRNA^Gly^ molecules from eukaryotic DTD1s, enabling successful mitochondrial translation. This co-evolution between DTD1 and tRNA^Gly^ also explains why opisthokonts, all fungi and animals, have only a single DTD1 enzyme that is colocalized to both mitochondria and cytoplasm (Figure 5) (37). Strikingly, this code transition could be captured in early branched eukaryotic organisms called Jakobids (37), whose mitochondria are closer to bacteria and possess one of the largest mitochondrial genomes (84). Indeed, they possess mitochondrial tRNA^Gly^ with both adenine and uracil at the discriminator base position, suggesting that the switch to only A73 occurred after the emergence of Jakobids. Jakobids eventually lost the U73 containing tRNA^Gly^ and the A73 possessing one was selected thereafter in all opisthokonts (Figure 5) (37).

### Plants do it differently

Plants, which represent another major eukaryotic branch of life, did not resort to the organellar discriminator code switch that was found in the opisthokonts (38), even though their DTD1 is of eukaryotic type. Therefore, our recent investigations focused on how the plant branch of life resolved the discriminator code conflict (38). Plants have devised a unique solution to DTD1-discriminator base conflict where DTD1 is absolutely restricted to the cytosol (Figure 5). Any deviation from this absolute restriction results in cross-compartment lethality. As mentioned earlier, plants possess archaeal origin DTD2 in addition to DTD1. Interestingly, plants have targeted archaeal-derived DTD2 to both the organelles, mitochondria and chloroplast, to serve as organellar chiral proofreader due to its strict D-chiral selection mode of operation. Therefore, such a mutual exclusion of conflicting components is essential for sustaining protein biosynthesis in plants (Figure 5) (38). It's interesting to note that plants have restricted the bacterial-derived DTD1 to function in the archaeal-derived cytosolic compartment whereas archaeal DTD2 is directed to bacterial-derived organelles. Overall, our studies have highlighted the importance of the optimization events of both DTD and its discriminator code in the two major branches of eukaryotic life that were critical for their emergence as well as evolution (Figure 5) (37,38).

### The discriminator code appears to be rooted deeper in translation

**Figure 6.**
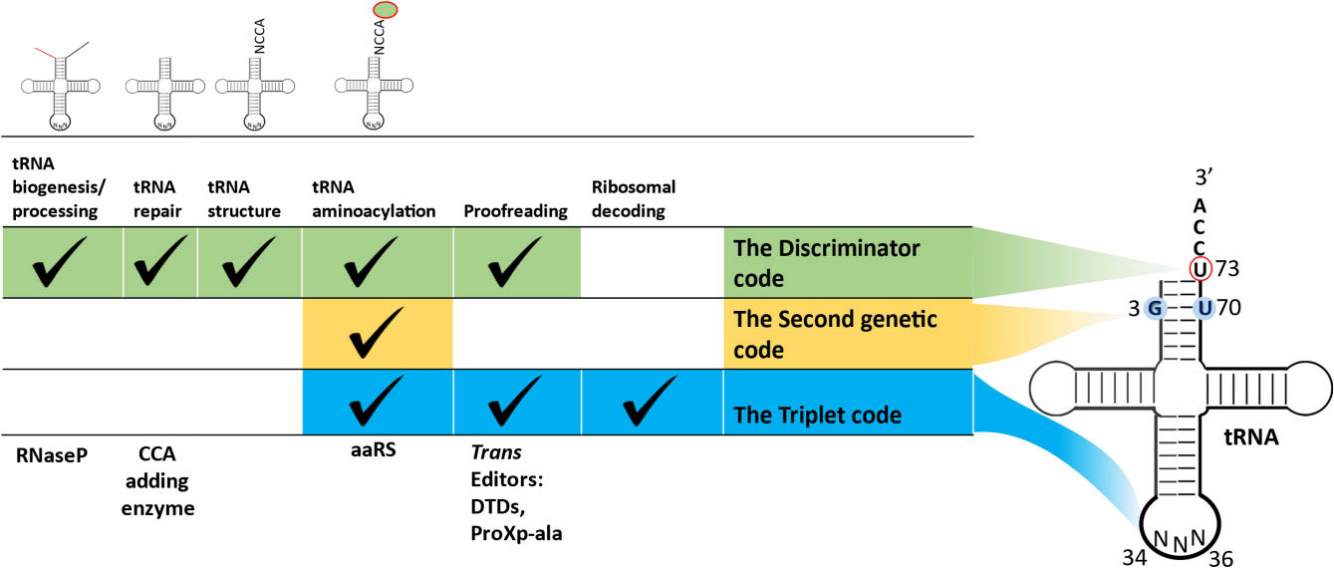
Discriminator base is deeply rooted in translation apparatus. Figure depicting the role of discriminator base (21,37,38,92–95), G3•U70 wobble base pair (78,79) and anticodon (14,82) throughout the translation process.

Biological life came into existence about 3.8 billion years ago after chemical evolution. Ribose, an integral part of ribonucleic acid (RNA), formed via a formose reaction (85,86). However, the exact reaction that led to the formation of RNA is still unclear. Once emerged, RNA-based chemistry might have ruled the early translation apparatus (87–89) where tRNA is thought to be evolved in a step wise manner from single nucleotide to tetranucleotide to microhelix to mini helix to fully functional tRNA (90,91). Identity of nucleotides could have been instrumental in providing specificity for amino acids at a very early stage. Therefore, as proposed by Crothers, discriminator base could have been a primordial code for amino acid selection in primordial apparatus (72) whereas other determinants like anticodons were probably later additions that expanded the amino acid gamut in translation apparatus. Newly evolved proteins developed specificity towards available RNA molecules and the discriminator code might have been a tool for the major transitioning from an RNA-based world to RNA-protein based hybrid world (35,36). A careful analysis of three key tRNA determinants (discriminator base, G•U wobble pair and anticodon) used throughout protein synthesis process shows the integration of discriminator based regulation at every step starting from tRNA biogenesis (Figure 6). Discriminator base modulates the biochemical activity of RNaseP (tRNA biogenesis) (92), CCA adding enzyme (tRNA biogenesis and repair) (93), tRNA structure (94), aaRSs (aminoacylation) (82) and *trans* editors like DTD1 and ProXp-ala (translation proofreading) (21,37,38,95), wherein G•U wobble pair is involved in aminoacylation (78,79) and anticodon dictates aminoacylation, proofreading and ribosomal decoding activities (14,82) (Figure 6). tRNA also evolved to have a plethora of modifications which are known to play crucial roles and control multiple physiological processes. Intriguingly, modifications are reported throughout the tRNA body except for CCA end and discriminator base (96). Discriminator base shows striking kingdom specific conservation for specific nucleotide bases in the tRNA molecules (38) that makes it an ideal candidate to impart specificity in enzymes. On the other hand, only the 34th nucleotide in anticodon (34th, 35th and 36th nucleotides) shows mutually exclusive dichotomy between eukaryotes and prokaryotes. The eukaryote four-box tRNAs contain exclusively an A at position 34 (the G is forbidden) whereas the prokaryotic counterpart contains a G34 (the A is forbidden) (97–99). Expression of G34 containing tRNAs induce miscoding and are toxic to the eukaryotic cells (100). It is interesting to note that tRNA^Gly^ stands as the only exception to this rule in the sense that both eukaryotes and prokaryotes tRNA^Gly^ contain G34 (101). Overall, the significant role of discriminator base throughout translation apparatus shows the critical role played by the ‘Discriminator code’ in the evolution of translation apparatus.

### Paul Berg meets Donald Crothers

**Figure 7.**
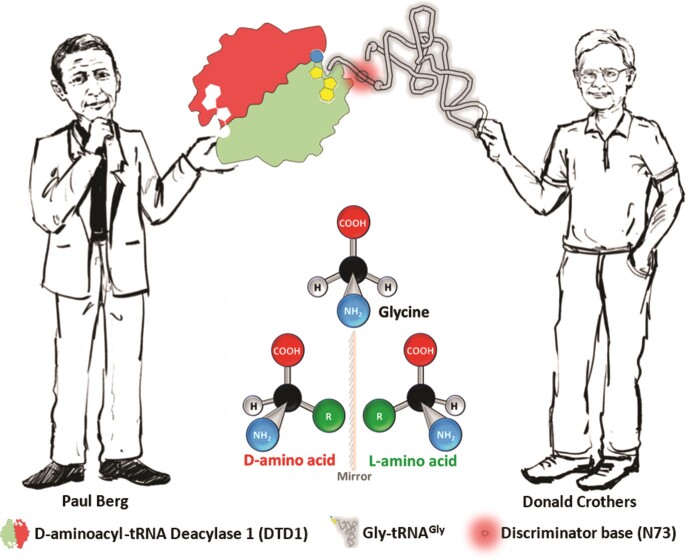
Paul Berg meets Donald Crothers. Figure depicting achiral glycine specific tRNA being unique to both DTD1 activity and discriminator hypothesis, bringing together the findings of two stalwarts of modern molecular biology (Caricature by Dr Sudipta Mondal, CSIR-CCMB, India).

Berg and Crothers had independent scientific journeys. Their identification of a ‘chiral proofreader’ and the proposal of a ‘discriminator code hypothesis’, respectively, are approximately half a century old (17,72). In both of these cases, achiral glycine and its tRNA being outliers, were neglected for long. Indeed, the acceptance of the universality of the D-amino acids across organisms is a relatively recent phenomenon and current efforts are further highlighting how they are handled across systems, including humans (102). Therefore, it took nearly half a century to identify and fully appreciate the hidden connection between the early work of Paul Berg and Donald Crothers, with their paths intersecting *via* an achiral bridge in protein biosynthesis (Figure 7).

## Conclusion and perspective

Fidelity during translation of genetic code is crucial for the survival of living systems. DTD1 is a unique *trans* acting editing module that proofread both chiral error as well as near-cognate error (19). The unpreceded role of discriminator base (N73) in tRNA acting as anti-determinant is essential to avoid cognate Gly-tRNA^Gly^ from DTD1’s unwanted activity (21). Modulating DTD1 activity through tRNA elements ensures that glycine is delivered faithfully to the ribosomal machinery during protein biosynthesis. Rewiring of both DTD1 and tRNA discriminator code enabled the evolutionary transition of bacteria into successful organelles (37,38). Also, the DTD-tRNA-code was optimized via two unique mechanisms in two major branches of eukaryotes, opisthokonts and plants, in order to avoid misediting of Gly-tRNA^Gly^ for uninterrupted translation in both cytosol and organelles crucial for their emergence and evolution (37,38).

Our studies underscore the necessity to probe optimization of cellular processes during major evolutionary transitions such as eukaryogenesis, as it brings together organisms that have independently evolved over two billion years. In addition, the ability to sequence whole genomes of organisms with cost effective methodologies and minimal sample requirements, is allowing us to look at many intermediates in evolution of traits. Over the last decade, a multitude of genomic studies across life forms have identified that key evolutionary jumps are associated with the removal or expansion of specific gene pool in addition to acquiring or innovating new processes. For example, land plant evolution has witnessed multiple episodes of horizontal gene transfers for diverse physiological and developmental processes that allowed their transition from unicellular simpler algal form to multicellular complex organism and their survival on the land (103,104). In similar lines, the genomic features specific to different lineage of opisthokonts originated even before their emergence (105,106). Overall, these cross genome analysis studies are revealing surprising evidences that not only help us in understanding how new traits are acquired, but also how existing traits are optimized to produce the evolutionary jump in both plant and animal branches of life (104,107). Multiple studies are now emerging which are identifying new codes dictating various aspects of biological evolution. The theme of ‘Code biology’ as proposed by Marcello Barbieri is becoming one of the most relevant aspects to consider in the context of biological evolution (108).

Chiral proofreading function is universally conserved but not the DTD protein. As mentioned earlier, three non-homologous proteins (DTD1, DTD2 and DTD3) of independent origins are identified till date which are implicated in chirality based proofreading in a domain specific manner. It is puzzling to note that life has opted to invent majorly two totally independent paths i.e. DTD1 in bacteria and DTD2 in archaea, to solve the fundamental problem of enforcing homochirality during protein biosynthesis. This could be a result of one of the following possibilities: (a) the chiral proofreading activity might not be essential for the emergence of life; (b) chiral proofreading was essential for the emergence of life and it is yet to be identified or (c) a primordial chiral proofreader was present in LUCA that was lost during the bifurcation of bacteria and archaea with the appearance of chiral proofreaders like DTD1 and DTD2, which have additional activities such as non-cognate and adduct removal activities, respectively (19,20,25).

Overall, the findings of Paul Berg and Donald Crothers intersected on an important aspect of protein biosynthesis i.e. chirality-based selection mechanisms during proofreading of the translation of the genetic code. This connection is established after the careful analysis of outliers (21,37,38) observed half a century ago by two independent groups, Paul Berg and Donald Crothers, working on two totally different aspects of protein synthesis (17,72). Paul Berg was working to identify d-amino acid removal system (17) while Donald Crothers was interested in understanding how amino acids were selected at an early stage of life (72). None of these two groups or others pursued and discussed the outliers in their future research. This type of connection is very rare in biological research, and, in fact, we have not seen any such intersections through outliers to the best of our knowledge. The scenario discussed in this manuscript once again highlights an important aspect of research i.e. excellent work, even if not noticed when done, has a way of surfacing again. An underlying message from the pioneering scientists, Berg and Crothers, is the unbiased way of reporting so called ‘outliers’ to their main findings despite not being the focus of work. It has become a rarity in reporting nowadays since either the authors remove it on their own or fearing the reviewers!

## Data Availability

No new data were generated or analysed in support of this research.
